# Human IgG/FcγR Interactions Are Modulated by Streptococcal IgG Glycan Hydrolysis

**DOI:** 10.1371/journal.pone.0001413

**Published:** 2008-01-09

**Authors:** Maria Allhorn, Anders I. Olin, Falk Nimmerjahn, Mattias Collin

**Affiliations:** 1 Division of Infection Medicine, Department of Clinical Sciences, Lund University, Lund, Sweden; 2 Experimental Immunology and Immunotherapy, Nikolaus-Fiebiger-Center for Molecular Medicine, Erlangen, Germany; Columbia University, United States of America

## Abstract

**Background:**

The human pathogen *Streptococcus pyogenes* produces an endoglycosidase, EndoS that hydrolyzes the chitobiose core of the asparagine-linked glycan on the heavy chain of human IgG. IgG-binding to Fc gamma receptors (FcγR) on leukocytes triggers effector functions including phagocytosis, oxidative burst and the release of inflammatory mediators. The interactions between FcγR and the Fc domain of IgG depend on the IgG glycosylation state.

**Methodology/Principal Findings:**

Here we show for the first time that EndoS hydrolyzes the heavy chain glycan of all four human IgG subclasses (IgG1-4), in purified form and in a plasma environment. An inactive form of EndoS, obtained by site-directed mutagenesis, binds IgG with high affinity, in contrast to wild type EndoS that only transiently interacts with IgG, as shown by Slot-blotting and surface plasmon resonance technology. Furthermore, EndoS hydrolysis of the IgG glycan influences the binding of IgG to immobilized soluble FcγR and to an erythroleukemic cell line, K562, expressing FcγRIIa. Incubation of whole blood with EndoS results in a dramatic decrease of IgG binding to activated monocytes as analyzed by flow cytometry. Moreover, the IgG bound to K562 cells dissociates when cells are treated with EndoS. Likewise, IgG bound to immobilized FcγRIIa and subsequently treated with EndoS, dissociates from the receptor as analyzed by surface plasmon resonance and Western blot.

**Conclusions/Significance:**

We provide novel information about bacterial enzymatic modulation of the IgG/FcγR interaction that emphasizes the importance of glycosylation for antibody effector functions. Moreover, EndoS could be used as a biochemical tool for specific IgG *N*-glycan hydrolysis and IgG purification/detection, or as a potential immunosuppressing agent for treatment of antibody-mediated pathological processes.

## Introduction

**Figure 1:**
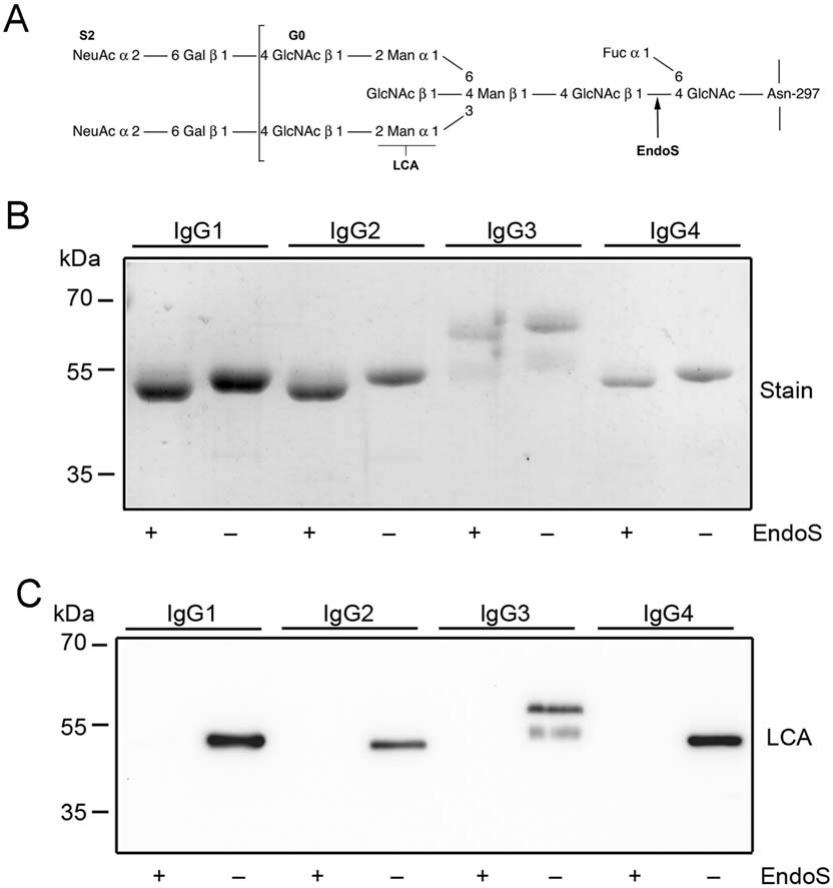
EndoS has glycosidase activity on all four human IgG subclasses. Panel A. Glycan structure of human IgG. Glycan on the γ−chains of IgG attached to aspargine 297. GlcNAc, *N*-acetylglucosamine; Fuc, fucose; Man, mannose; Gal, galactose; NeuAc, sialic acid. Cleavage site for EndoS and recognation site for *Lens culinaris* agglutinin lectin (LCA) are indicated. Panel B. Purified IgG 1–4 incubated with EndoS and analyzed by SDS-PAGE and stained. Panel C. IgG 1–4 incubated with EndoS and analyzed using LCA lectin blot.

The immunoglobulin G (IgG) class of antibodies plays an important role in the adaptive immune defense of the human host against pathogens. IgG consists of two identical heavy chains and two identical light chains, which in turn are composed of variable and constant domains. Papain treatment of the IgG molecule generates two separate monovalent Fab fragments recognizing antigens and an intact Fc fragment, a recognition site for host receptors and a site of interaction with a number of effector molecules, including the classical complement pathway starting with factor C1q [1], [2]. IgG is a glycoprotein containing a conserved complex carbohydrate structure attached to the asparagine 297 residue in the CH2 domain of each IgG Fc part. It consists of a biantennary core of *N*-acetylglucosamine and mannose with added terminal and branching carbohydrate residues such as *N*-acetylglucosamine, fucose, sialic acid, and galactose (Fig. 1A) [3]. The presence of this carbohydrate is crucial for proper antibody structure and for interactions with cellular immunoglobulin G Fcγ receptors (FcγRs) and the complement system [1], [4]. Altered glycosylation of IgG have been associated with autoimmune disorders like rheumatoid arthritis (RA), systemic lupus erythematosus (SLE) and Crohńs disease [5]–[7]. Several forms of autoimmune vasculitis show a differentiating glycosylation with decreased Fc galactosylation and sialylation [8]–[12]. Additionally, it has been suggested that sialylated IgG *in vivo* switch from its anti-inflammatory activity with subsequent reduced antibody effector activity, to a pro-inflammatory/toxic activity upon decreased Fc sialylation [13]. IgGs, classified into four subclasses, IgG1, IgG2, IgG3 and IgG4, are described to interact with different types of FcγRs giving different activation profiles [14], [15].

FcγRs provide a linkage between the humoral and cellular immune responses. Phagocytic cells express members of three classes of IgG-Fc receptors, FcγRI, FcγRII and FcγRIII, characterized by structural and functional homology and by the specific recognition site on the CH2 region of IgG [1], [16], [17]. Binding of pathogen-IgG complexes to FcγRs mediates an essential response from the host against pathogens by initiating a cascade of signals causing antibody-dependent-cellular-cytotoxicity (ADCC), complement-dependent-cellular-cytotoxicity (CDCC), endocytosis, phagocytosis, oxidative burst, the release of inflammatory mediators, etc. [2], [18]. Complexed IgG-FcγR can besides activation of the C1q component of complement also activate other ligands e.g. mannan binding lectin (MBL), the neonatal receptor FcRn, the mannose receptor (MR), etc. [2], [4]. FcγRs may be expressed constitutively on haematopoietic cells and may also be induced or up-regulated by cytokines and other agents. FcγRs are responsible for balancing activation (FcγRI, FcγRIIa and FcγRIIIa) and inhibitory signals (FcγRIIb) of the immune system with the ability of both activating and inhibiting the IgG mediated effector stimulation [1], [19].

*Streptococcus pyogenes* is one of the most common human pathogens causing pharyngitis, scarlatina and more severe infections like necrotizing fasciitis and sepsis [20], [21]. Like other bacteria it expresses several virulence factors and uses several immune evasion strategies to successfully invade its host [22]–[25]. The recently discovered enzyme Endoglycosidase S (EndoS) is secreted by *Streptococcus pyogenes* and has a specific endoglycosidase activity on native IgG by hydrolyzing the conserved asparagine-linked glycans on the heavy chains of IgG (Fig. 1A) [24], [26]. This 108 kDa-enzyme is encoded by the gene *ndoS* that is highly conserved and is present in virtually all examinated isolates. EndoS is the first known bacterial enzyme with a unique specificity for native IgG [24]. This is in contrast to other related endoglycosidases as EndoF1-3 from *Elisabethkingia meningoseptica* (previously *Flavobacterium meningosepticum*), which show enhanced hydrolytic activities on the denaturated forms of basically any glycoproteins with the appropriate N-linked glycan, or EndoE from *Enterococcus faecalis* that in addition to activity on the glycan of native IgG also hydrolyzes high-mannose glycans on other denatureted glycoproteins [27], [28]. EndoS is N-terminally processed by the cysteine proteinase SpeB that could be of importance in regulating EndoS activity [29]. Furthermore, the molecular requirements for EndoS glycosidase activity have recently been elucidated revealing the importance of glutamic acid 235 (Glu-235) and tryptophans [29]. EndoS activity affects the functionality of opsonizing IgG by decreased binding to Fc-receptors on a monocyte-like cell line and impaired classical complement activation *in vitro* [26].

In the present study we elucidated the effect(s) of EndoS on IgG subclasses and IgG-FcγR interactions. The results revealed that EndoS hydrolyses the heavy chain of all four human IgG subclasses (IgG1–4), both soluble and in a plasma environment. Additionally, we found that EndoS hydrolysis of the IgG glycan dramatically influences the binding of IgG to soluble, immobilized FcγRIIa and FcγRIIb as well as to FcγR-expressing cells. Moreover, IgG pre-bound to these cells dissociates due to treatment of cells with EndoS. Furthermore, an inactive form of EndoS generated by site-directed mutagenesis binds with high affinity to IgG1–4, while the active form only transiently interacts with its substrates. These results provide novel information about the mechanisms behind enzymatic modulation of the host immune defense by bacteria, provide novel information about the molecular interactions between an IgG glycan-hydrolyzing enzyme and IgG, and emphasize the importance of IgG glycosylation for correct antibody effector functions.

## Results

### EndoS has glycosidase activity on all four IgG subclasses

**Figure 2:**
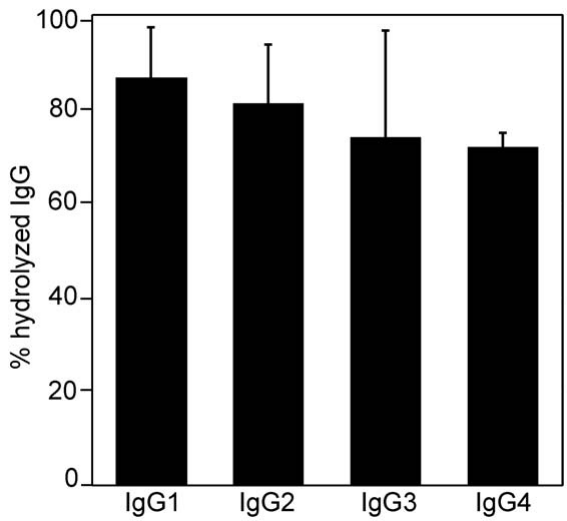
Hydrolysis of human IgG-subclasses by EndoS in plasma environment. Human plasma treated with EndoS or PBS and followed by IgG glycan hydrolysis detection using LCA lectin ELISA of the purified IgG fraction. The results are presented as percent hydrolysis of each subclass compared to signals from untreated plasma. Means and standard deviations, indicated with error bars, were calculated from three independed experiments using blood from three different donors.

It has previously been shown that EndoS hydrolyzes the chitobiose core of the conserved N-linked glycan on the γ-chain of human polyclonal IgG [24] (Fig. 1A). It was therefore of interest to elucidate whether EndoS has activity on all four subclasses of human IgG (IgG1–4). Purified recombinant EndoS was incubated with purified human IgG1–4. SDS-PAGE analysis revealed that EndoS-treated IgG of all subclasses migrated at an apparent molecular weight of approximately 3 kDa lower than untreated IgG (Fig. 1B), which is consistent with hydrolysis of the chitobiose core of the IgG glycan. To confirm glycan hydrolysis, samples were also analyzed by lectin blot using a *Lens culinaris* agglutinin (LCA) lectin recognizing α-linked mannose residues (Fig. 1A). Lectin blot analysis of the samples revealed that all IgG subclasses lose the reactivity with LCA after incubation with EndoS consistent with complete or nearly complete hydrolysis of the glycan (Fig. 1C). Additionally, the glycosidase activity of EndoS on IgG1–4 in a plasma environment was investigated. In this experiment human plasma was incubated with purified EndoS or buffer, followed by affinity purification of the IgG fraction. These fractions were subsequently subjected to a LCA ELISA using immobilized monoclonal antibodies against IgG1–4 to capture IgG. This revealed that all four IgG subclasses reacted with lectin when plasma was incubated with buffer, indicating presence of the glycan (data not shown). In contrast, when plasma was treated with EndoS, a dramatically reduced IgG1–4 reactivity with LCA lectin was observed. IgG1 was hydrolyzed to 87±11%, IgG2 was hydrolyzed to 81±13%, IgG3 was hydrolyzed to 74±23%, and IgG4 was hydrolyzed to 72±3% (Fig. 2). Taken together, these results clearly show that EndoS has the ability to hydrolyze human IgG of all subclasses, in purified form as well as in whole plasma.

### The inactive form of EndoS binds IgG

**Figure 3:**
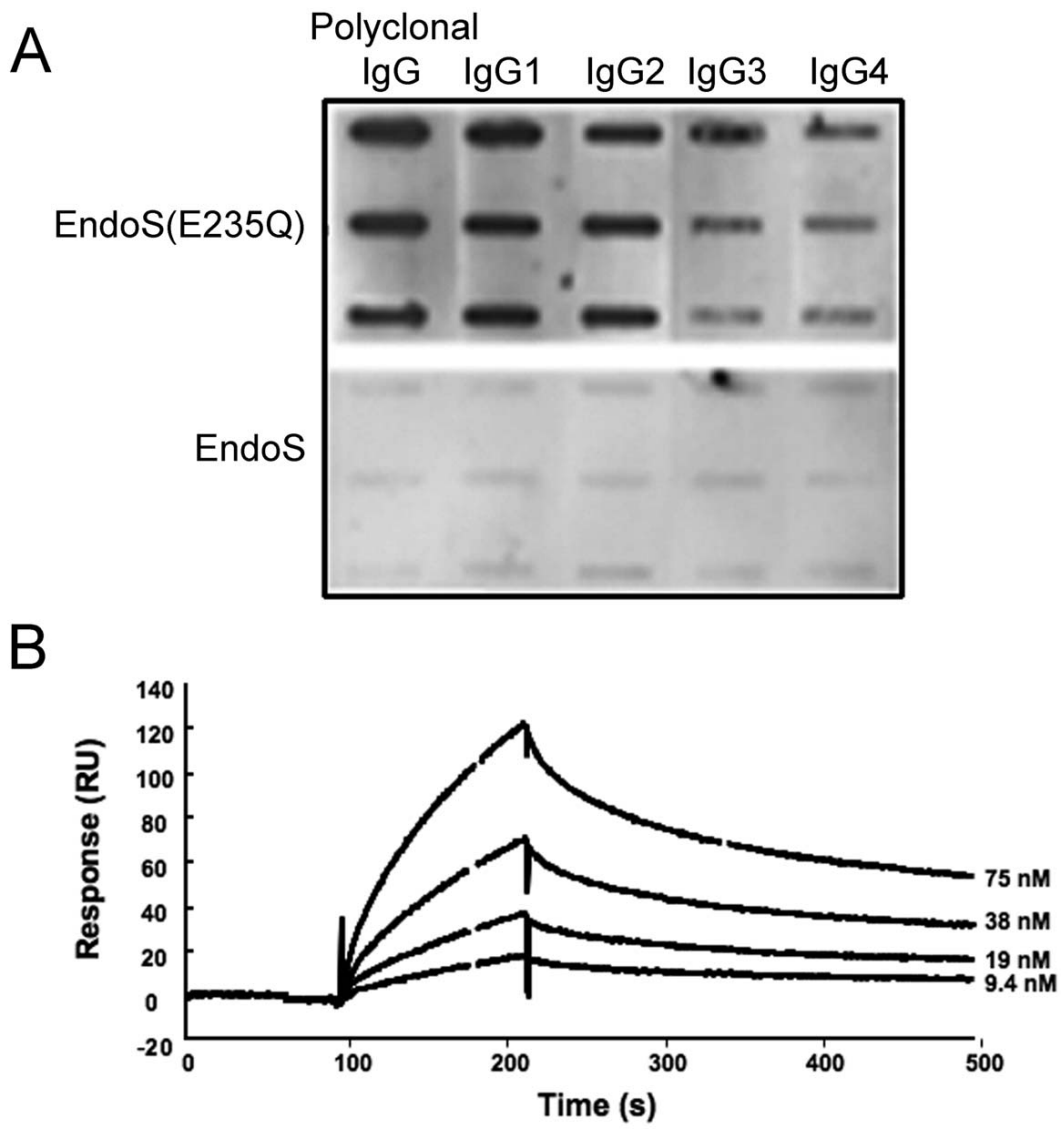
EndoS(E235Q) binds to all IgG-subclasses. Panel A. Slot-blot representing the binding of EndoS and EndoS (E235Q) to each IgG subclass immobilized onto a nitrocellulose membrane in amounts: 3, 1.5 and 0.75 µg. The binding was detected using antiserum against EndoS. Panel B. Binding of EndoS (E235Q) to immobilized IgG-classes using BIAcore technology. The selected plot shows EndoS (E235Q) binding to IgG1 using hydrolysed IgG1 as a reference (bulk changes subtracted).

We have previously partly elucidated the molecular requirements for EndoS hydrolysis of IgG. Site directed mutagenesis of glutamic acid 235 to glutamine (EndoS(E235Q)) at the proposed orifice of the catalytic tunnel abolishes enzymatic activity. In addition, chemical blocking of tryptophanes revealed that these amino acid residues are important for activity [29]. To further investigate the physical interaction between enzyme and substrate, the binding of EndoS and EndoS(E235Q) to immobilized polyclonal IgG and IgG1–4 subclasses was studied using slot-binding experiments with immobilized IgG probed with EndoS and EndoS(E235Q). Purified, soluble IgG subclasses 1–4, each immobilized onto a nitrocellulose membrane, were probed with EndoS and EndoS(E235Q) followed by incubation with antibodies against EndoS. This experiment revealed a strong binding of EndoS(E235Q) to polyclonal IgG, IgG1 and IgG2, and a weaker association to IgG3 and IgG4, while only very weak interactions between active EndoS and all subclasses could be seen (Fig. 3A). To calculate the affinity constants between EndoS and immobilized IgG1–4, surface plasmon resonance technology was used. Similarly to slot-binding results, this showed that EndoS(E235Q) binds all IgG subclasses with high affinity, while there is no detectable binding of EndoS to IgG. (Fig. 3B, Table 1). The kinetic parameters of EndoS (E235Q) binding to immobilized IgG subclasses were of similar character and the strongest interaction was demonstrated between IgG1 and EndoS(E235Q) with a binding affinity constant (*K_D_*) of 0.42 µM. No binding of either EndoS or EndoS(E235Q) to IgG1–4 subclasses, which were hydrolysed by EndoS before immobilization, was detected. These findings indicate that the intact IgG glycan is necessary for the interaction between EndoS and IgG. Furthermore, the experiments comparing the interactions between EndoS, EndoS(E235Q) and IgG indicates that EndoS binds IgG with a high affinity, but the active enzyme is instantly released after glycan hydrolysis in a “touch and go” manner.

**Table 1:** Kinetic constants of EndoS binding to immobilized human IgG1–IgG4.

	EndoS (E235Q)	EndoS (E235Q)	EndoS (E235Q)	EndoS
	*k_a_* (×10^4^/M/s)	*k_d_* (×10^−3^/s)	*K_D_* (×10^−7^M)	
IgG1	4.2	17.6	4.2	nb^a^
IgG2	1.2	2.5	2.1	nb
IgG3	10.7	8.9	0.83	nb
IgG4	1.5	2.0	1.4	nb
IgG1–4^b^	nb	nb	nb	nb

a nb = no binding

b IgG1–4 hydrolyzed by EndoS

### EndoS influences the binding of IgG1–4 to FcγRs

**Figure 4:**
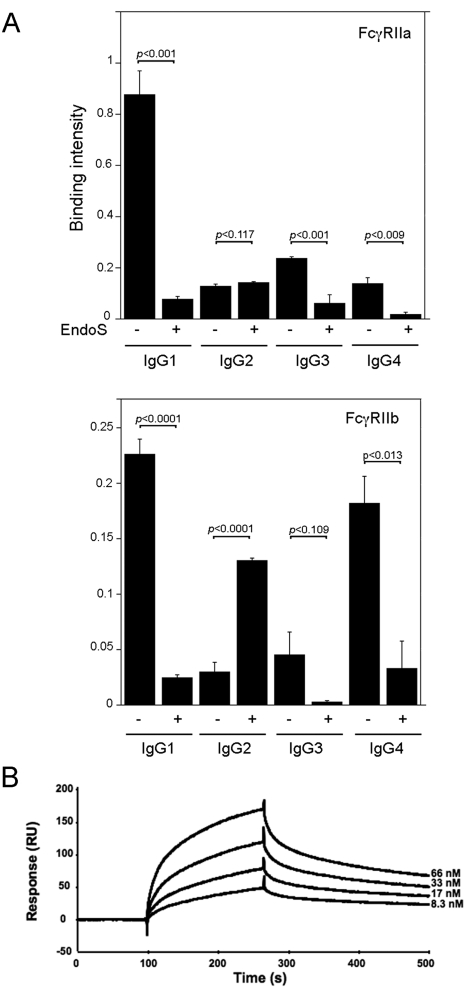
EndoS treatment of IgG subclasses inhibits binding of IgG to FcγRII. Panel A. Binding of purified IgG subclasses, with or without EndoS treatment, to FcγRIIa and FcγRIIb immobilized to a microtitter plate. HRP-labeled protein G was used for detection of bound IgG subclasses. (−) indicates intact IgG and (+) EndoS hydrolysed IgG. Means, standard deviations (indicated with error bars), and *p* values (calculated using Student's *t*-test) were determined from three separate experiments. Panel B. Binding of IgG subclasses to immobilized receptors as visualized using BIAcore surface plasmon resonance. Plot shows a typical sensorgram, here the IgG1 binding to FcγRIIa. An empty flow cell is used as reference (subtracted).

Since the nature of the interactions between FcγRs and the Fc domain of IgG is highly dependent on the IgG glycosylation state [1], [3], [4] we explored the effects of EndoS activity on IgG interactions with FcγRs. Thus, in an ELISA experiment the soluble FcγRIIa, FcγRIIb and FcγRIIIa were immobilized and probed with purified IgG1–4 subclasses. In line with other observations it was here seen that FcγRIIa and FcγRIIb binds IgG1 [14]. This binding was nearly abolished after treatment of IgG1 with EndoS. The binding of the other IgG subclasses to these receptors was weak and was even more reduced after treatment with EndoS. In general, ELISA studies revealed the IgG subclass binding affinity pattern IgG1>IgG3>IgG4>IgG2 for FcγRIIa and IgG1>IgG4>IgG3>IgG2 for FcγRIIb (Fig. 4A). Furthermore, we observed that the EndoS hydrolysed IgG2 had a different outcome regarding the binding to FcγRIIa/FcγRIIb with more extensive binding ability, compared to the untreated IgG2. FcγRIIIa was negative in binding of all IgG subclasses (data not shown). The interaction between IgG1–4, with or without EndoS treatment, with FcγRs was further analyzed by surface plasmon resonance. Each IgG subclass was tested for binding to a surface with an immobilized FcγRIIa, FcγRIIb or FcγRIIIa. Consistent with the ELISA data, the results showed that IgG1 had the strongest affinity for both FcγRIIa and FcγRIIb with similar binding affinity constants, 97 nM and 170 nM respectively (Fig. 4B, Table 2). In agreement with our previous findings, no binding of IgG1 to these receptors was detectable when EndoS treated IgG1 was used. There was no detectable interaction between FcγRIIa/FcγRIIb and IgG2 or IgG3, or between IgG4 and FcγRIIa (Table 2). No binding of soluble IgG1–4 subclasses to immobilized FcRIIIa could be detected. These results indicate that EndoS hydrolysis dramatically decreases IgG's affinity for FcγRs.

**Table 2:** Kinetic constants of IgG1–IgG4 binding to different Fc-receptors.

	FcγRIIa	FcγRIIa	FcγRIIa	FcγRIIb	FcγRIIb	FcγRIIb	FcγRIIIa
	*k_a_* (×10^4^/M/s)	*k_d_* (×10^−3^/s)	*K_D_* (×10^−7^M)	*k_a_* (×10^4^/M/s)	*k_d_* (×10^−3^/s)	*K_D_*(×10^−7^ M)	
IgG1	3.5	3.4	0.97	2.6	4.3	1.7	nb^a^
IgG2	nb	nb	nb	nb	nb	nb	nb
IgG3	nb	nb	nb	nb	nb	nb	nb
IgG4	nb	nb	nb	3.4	6.9	2.0	nb
IgG1–4^b^	nb	nb	nb	nb	nb	nb	nb

a nb = no binding

b IgG1–4 hydrolyzed by EndoS

### EndoS decreases IgG binding to blood cells

**Figure 5:**
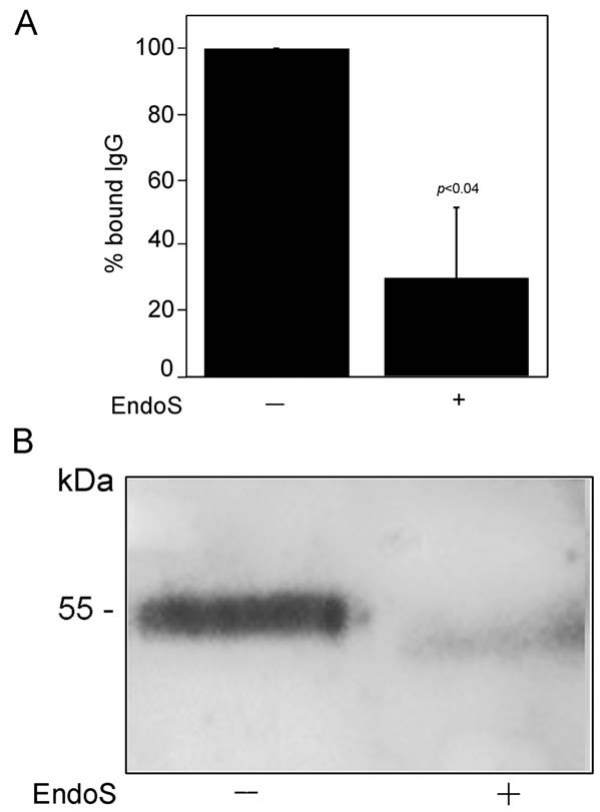
EndoS treated IgG does not bind to FcγRIIa on K562 cells. Panel A. The relative binding of radioactive IgG, With or without EndoS treatment, to K562 cells. The cells were incubated with ^125^iodine-labelled IgG (intact or EndoS-treated). The radioactivity of the washed cell pellets was detected. The binding of ^125^I- IgG (intact) to K562 cells, presented here as 100%, represents a specific IgG binding to K562 cells that could be inhibited by addition of cold IgG. Means, standard deviations (indicated with error bars), and *p* values (calculated using Student's *t*-test) were determined from three separate experiments. Panel B. K562 cells were incubated with human plasma treated with EndoS or PBS. The cells were resuspended in lysis buffer and analyzed by SDS-PAGE and Western blot using antiserum against human IgG.

**Figure 6:**
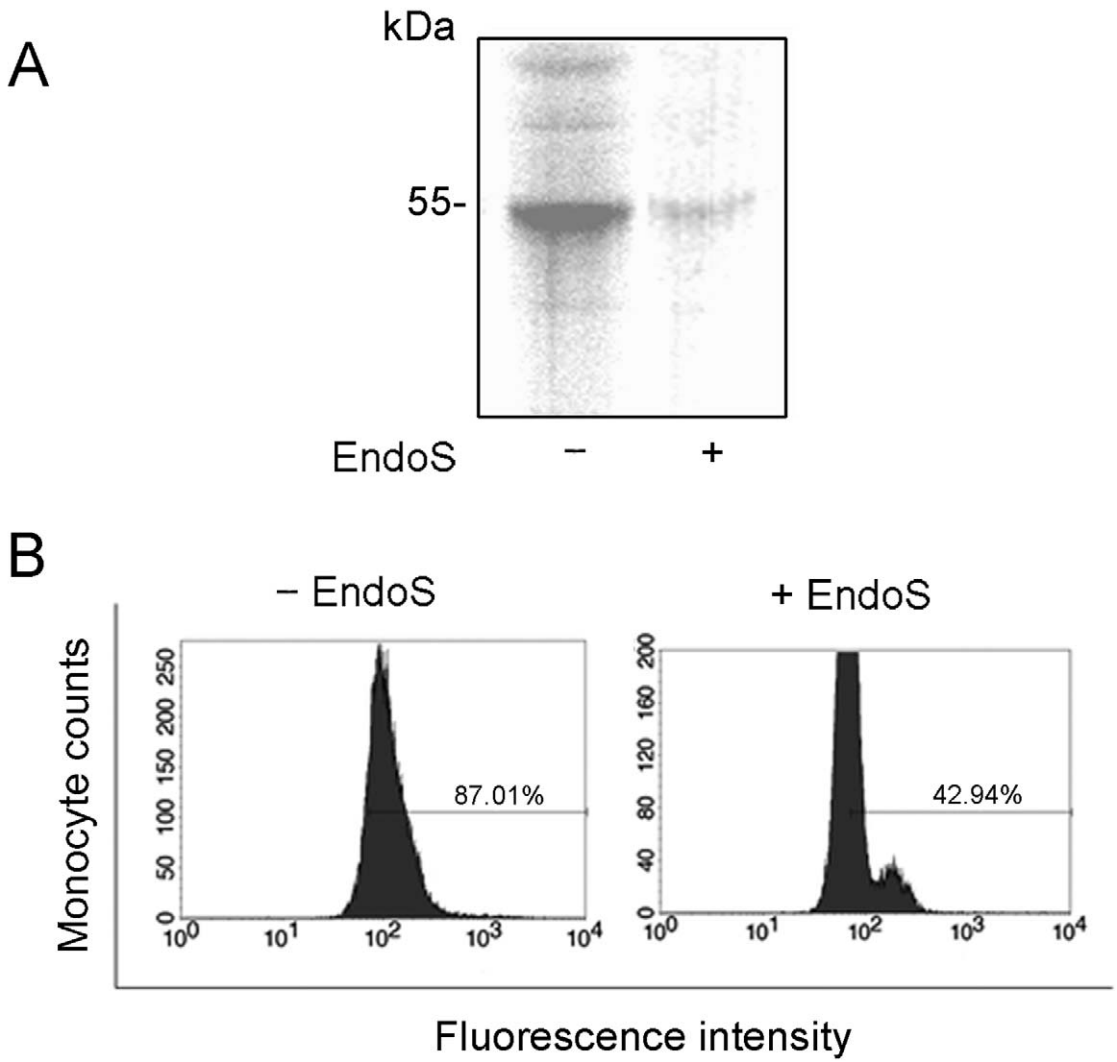
EndoS treated IgG does not bind to monocytes. Panel A. Monocytes were incubated with ^125^iodine-labelled IgG (intact or EndoS-treated). After incubation for 30 minutes at room temperature, the proteins from cell lysates, 10 µg total protein, were separated by 10% SDS-PAGE. The gel was dried and analyzed by phosphorimaging. Panel B. Flow cytometry analysis showing the decreased binding of IgG to activated monocytes in blood treated with EndoS. Human blood was treated with EndoS before addition of leukocyte activator fMLP. The IgG binding to monocytes was detected using mouse anti-human IgG and FITC-labelled goat anti-mouse IgG as a secondary antibody.

Based on results from ELISA and surface plasmon resonance, we continued to analyze the effect(s) of EndoS glycosidase activity on the interaction between FcγRs and IgG. For this purpose we used an erythroleukemic cell line (K562) exclusively expressing FcγRIIa [14], [30]. Since soluble FcγRI was not available to us, we also investigated human monocytes that predominantly bind IgG through this receptor [14]. Thus, IgG was purified from plasma treated with EndoS or PBS, labeled with ^125^I and incubated with the K562 cells. The radioactivity of the cell pellets was measured. This revealed significantly decreased binding of radioactive IgG, originally purified from plasma treated with EndoS, to K562 cells (Fig. 5A). In a control experiment, the specific IgG binding to these cells was calculated by addition of cold human IgG, which inhibited the binding of radioactive IgG to 93% (data not shown). A strong binding of IgG to K562 cells after incubation of cells with human plasma was confirmed by Western blot and the reactivity of cell lysates with antibodies against human IgG. In contrast, there was a clear decrease in binding of IgG to K562 cells incubated with plasma pre-treated with EndoS (Fig. 5B). Likewise, the binding of ^125^I-IgG to monocytes as analyzed by SDS-PAGE was totally inhibited when IgG was treated with EndoS (Fig. 6A). To further analyze EndoS' influence on the interaction between FcγRs on monocytes and IgG, flow cytometry analysis of whole blood was performed. Human blood was pre-incubated with EndoS before addition of the leukocyte activator fMLP. Monocytes were gated based on forward and side scatter and the reactivity of monocytes with monoclonal anti-human IgG was evaluated. The result revealed that 87% of monocytes were positive for IgG binding, while only 43% of monocytes in blood incubated with EndoS were positive (Fig. 6B). These results indicate that EndoS-hydrolyzed IgG is significantly reduced in its binding capacity to human cells expressing different sets of FcγRs.

### IgG dissociates from FcγRIIa upon treatment with EndoS

**Figure 7:**
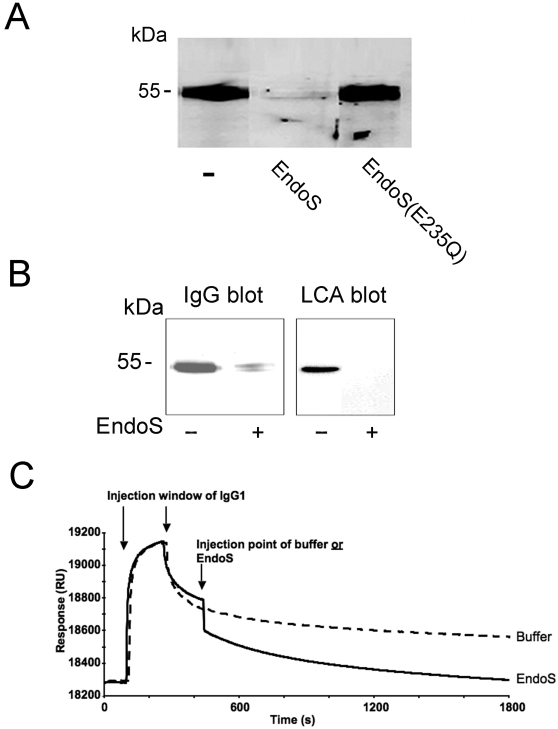
Dissociation of IgG from FcγRII upon treatment with EndoS. Panel A. IgG bound to K562 cells dissociates from FcγRIIa upon incubation with EndoS but not with EndoS(E235Q). K562 cells were incubated with plasma and subsequently with EndoS, EndoS(E235Q) or PBS. Cell lysates, 10 µg total protein, were analysed for IgG by SDS-PAGE and blot using antiserum against human IgG. Panel B. IgG bound to monocytes dissociates from FcγRs after treatment with EndoS. Monocytes were incubated with plasma and later with EndoS or PBS. Resuspended cell pellets were analyzed for IgG by blot using antiserum against human IgG. The glycan of IgG was detected by blot and reactivity with LCA lectin. Panel C. A BIAcore setup showing EndoS affecting the IgG1 dissociation from an immobilized receptor FcγRIIa. In two parallel experiments, the injection of EndoS (black curve) is compared to the injection of buffer (broken line) at the same time-point during the dissociation phase of the IgG1- FcγRIIa interaction.

Our experiments this far have revealed that EndoS hydrolysis of IgG inhibits binding to FcγRs on cells and surfaces, but it remained unclear if EndoS has activity on IgG already bound to FcγRs and if such activity could release the IgG bound to FcγRs. Therefore, we investigated the effects of EndoS on IgG bound to K562 cells that had been exposed to human plasma and subsequently treated with EndoS. The cell lysates were analyzed by SDS-PAGE and Western blot using an antibody against human IgG. There was a significant binding of IgG to K562 cells as judged by the results presented in figure 7A. Interestingly, no IgG-band was visible on a blot when cells were treated with EndoS, suggesting a total IgG dissociation from the cells (Fig. 7A). A control experiment, using EndoS(E235Q), revealed an IgG signal on the surface of K562 cells comparable to the untreated cells. These results strongly suggest that IgG dissociates from the cell surface due to N-glycan hydrolysis of IgG by EndoS. Similarly, the effect of EndoS on IgG bound to monocytes was analyzed. This showed that most of the monocyte-bound IgG dissociated from cells due to the treatment with EndoS as compared to untreated cells (Fig. 7B). As expected, monocytes treated with EndoS, in contrast to control cells, showed no reaction with the LCA lectin, indicating that the minute amounts of IgG remaining on the cells as detected in the IgG blot had most likely been hydrolyzed by EndoS (Fig. 7B). The results demonstrated above were further confirmed by surface plasmon resonance experiments. Soluble IgG1 and FcγRII receptor were chosen because of our earlier observation that IgG1 is the strongest binder of FcγRII. After binding of IgG1 to pre-immobilized FcγRIIa and reaching a steady-state dissociation phase, the IgG1 injection was aborted and replaced by EndoS injection or running buffer. This revealed that EndoS injection causes the dissociation of IgG1 from immobilized FcγRIIa receptor while the IgG1 dissociation from FcγRIIa was unaffected when adding running buffer (Fig. 7C). Taken together, these results clearly demonstrate that EndoS by IgG glycan hydrolysis can release IgG bound to FcγRs on cells and surfaces.

## Discussion

In the present study we attempted to elucidate the physical interaction between EndoS and IgG and the physiological relevance of EndoS IgG *N*-glycan hydrolyzing activity for IgG-FcγR interactions. We present for the first time that EndoS specifically acts as an endoglycosidase on all human IgG subclasses, both in purified form and in a plasma environment. As expected, there is a physical interaction between the enzyme and all IgG subclasses, that we successfully demonstrated using an enzymatically inactive, mutated form of EndoS. In this study we could not separately investigate the EndoS effects on the isolated binding of IgG to FcγRI. However, we observed that EndoS-hydrolyzed IgG did not bind to monocytes, and that there was a nearly complete dissociation of IgG from monocytes upon hydrolysis by EndoS. Since monocytes express FcγRI and this receptor has the highest affinity for IgG, we conclude that EndoS influences IgG binding even to FcγRI because the effects of EndoS observed must be predominantly due to involvement of FcγRI. EndoS seems to have an effect on both isotypes of FcγRII receptors, thus influencing both activating and inhibiting IgG mediated effector stimulation. Interestingly, IgG2 treated with EndoS, in opposite to what was observed for the other subclasses of IgG, showed increased binding to FcγRIIb, and slightly also to FcγRIIa, immobilized to microtiter plate. One possible explanation for this could be aggregation of IgG2 upon hydrolysis by EndoS leading to increased binding to FcγRs. However, no binding of IgG2 to FcγRs was detected using surface plasmon resonance which is in agreement with earlier publications [14]. This could be explained by the constant flow of IgG2 over the immobilized receptors in the case of surface plasmon resonance, in contrast to ELISA where IgG2 is allowed to aggregate and interact with the receptor.

The activity of EndoS on IgG has obvious benefits for *S. pyogenes* expressing this enzyme with potential modulation and/or evasion of an IgG-mediated response against the bacteria. We have previously demonstrated that EndoS treatment of human opsonizing IgG antibodies directed towards the cell-wall anchored M protein significantly enhances the bacterial survival in blood [26]. Therefore, in the context of an intact infecting *S. pyogenes,* EndoS is a potentially harmful molecule to the human host that contributes to the bacterial virulence. In contrast to this, the purified form of EndoS has substantial potential as a biotechnological tool and/or a therapeutical agent that could be beneficial for future experimental science and possibly also health care.

Our results reveal that EndoS possesses a capacity to inhibit the IgG binding to FcγRs and detach IgG bound to FcγRs on cell surfaces. We have recently been able to show that pre-treatment of arthritogenic antibodies abrogates development of arthritis in a mouse model of collagen-induced arthritis [31]. This suggests that EndoS may have potential for being further developed as a therapeutical agent in other antibody-mediated autoimmune disorders.

We suggest two principally different biotechnological uses of EndoS, one based on the IgG-glycan hydrolyzing activity of the wild-type enzyme, and the other based on the high affinity IgG-binding of EndoS(E235Q). The active enzyme could be used for *in vitro* treatment of whole blood or purified blood cells in order to remove IgG already bound to various FcγRs on these cells. This could facilitate the analysis of effects of specific IgG preparations added to the cells, regarding receptor binding and cellular activation, without the interference of pre-bound IgG. The inactive form of EndoS (EndoS(E235Q)) has a great potential as a specific IgG purification and detection tool. In this study we have demonstrated that EndoS(E235Q) interacts equally well with all subclasses of IgG. This is comparable to what can be seen for protein G, one of the major molecules currently used for IgG preparation and detection [32], but advantageous compared to protein A that does not bind IgG3 [33]. Protein A also binds IgM and IgA to a certain extent. We have previously shown that there is no interaction between EndoS and IgM or IgA [24]. Furthermore, we could show here that EndoS(E235Q) does not interact with IgG lacking its heavy chain glycans. This is in contrast to both protein G and protein A that bind IgG irrespective of its glycosylation state [24]. This could be especially important when only intact IgG with a certain functional effector region is required. When using currently available reagents like protein G, a second purification step using for instance a lectin column is required to obtain only the glycosylated fraction of IgG. This property of EndoS(E235Q) could be used in combination with for instance protein G to assess the glycosylation state and the functional quality of an IgG preparation.

In conclusion, EndoS is a bacterial immunomodulatory protein with a great potential. Our results provide novel information about bacterial pathogenesis, i.e. how the pathogens evade the immune system of the host organism by affecting the functions of IgG/FcγRs. Moreover, EndoS could be used as an important biochemical tool for specific IgG *N*-glycan hydrolysis and IgG purification/detection, or perhaps as a potential immunosuppressing agent that could be used to interfere with antibody-mediated pathological processes.

## Materials and Methods

### Proteins and reagents

Blood was drawn from healthy individuals and collected in heparin-containing tubes. Full-length EndoS with glutathione-S-transferase (GST) as a fusion was recombinantly expressed and purified from *Escherichia coli* harboring the plasmid pGEXndoS. When appropriate, the GST-tag was removed using Factor Xa as previously described [23]. The mutation of glutamic acid 235 of EndoS into glutamine was performed using QuickChange II Site-Directed Mutagenesis Kit according to manufacturer's instructions (Stratagene, La Jolla, CA) with following verification of the mutation by sequencing [29]. Soluble purified Fc-receptors were generated by co-transfection of CHO-K1(CHO) cells with pNT-neo-FcγRII or pNT-neo-FcγRIII plasmids with subsequent selection in 1 mg/ml genetecin [34]. IgG-subclasses were produced by transient transfection in 293T cells [34]. RPMI 1640 medium and Hanḱs balanced salt solution (HBSS) were from GIBCO, Paisley, U.K. All other reagents were purchased from Sigma-Aldrich unless indicated otherwise.

### EndoS-treatment of antibodies

Purified human IgG1–4 was hydrolyzed with GST-EndoS purified as previously described [23]. Enzyme/substrate molar ratio was 1∶20 in PBS and samples were incubated for 2 h at 37°C. GST-EndoS was removed from the samples by passing three times over a glutathione-Sepharose column (Amersham Biosciences, Uppsala, Sweden). 1 µg of treated and untreated IgG1–4 was separated on 10% SDS-PAGE followed by staining with Coomassie Blue or *Lens culinaris* agglutinin (LCA)-lectin (Vector Laboratories, Burlingame, CA, USA) blot analysis (se below).

### Treatment of human plasma with EndoS and purification of IgG

A volume of 2 ml human plasma was incubated with 20 µg EndoS or a PBS equivalent for 1.5 hours at 37°C. The IgG fraction was purified using Protein G Sepharose (GE Healthcare Bio-sciences AB, Uppsala, Sweden). Briefly, 200 µl Protein G Sepharose suspended 1∶1 in PBS (phosphate-buffered saline; 10 mM phosphate buffer, pH 7.4, 120 mM NaCl, 3 mM KCl) was added to plasma samples and incubated at 4°C for 2 hours or over night. After centrifugation for five minutes at 8000×*g*, the supernatant was discarded and the pellet washed three times with PBS. IgG was eluated with 0.1 M glycine pH 2.0 and neutralized with 1 M Tris-HCl pH 8.0. The IgG concentration was determined to 8 mg/ml using the Advanced Protein Assay (Cytoskeleton, Denver, CO, USA).

### Cell preparations

The K562 cell line was cultured in RPMI 1640 medium supplemented with Glutamax-I, 100 µg/ml antibiotics (penicillin and streptomycin) and 10% fetal calf serum at 37°C in an atmosphere containing 5% CO_2 _and 95% humidity. Nunclon flasks for cell culture were used (Nunc A/S, Roskilde, Denmark). Cells were cultured in a serum free medium for 20 hours before being used in experiments. Monocytes were isolated from human whole blood using the Polymorphprep kit (AXIS-SHIELD, Oslo, Norway) or Ficoll-Paque Plus (Amersham Biosciences, Uppsala, Sweden) according to instructions provided by the manufacturers. After isolation, the cells were counted and resuspended in PBS or RPMI-medium.

### Enzyme linked immunosorbent assay (ELISA)

For glycan detection, microtiter plates (NUNC, Roskilde, Denmark) were coated with 100 µL monoclonal mouse anti-human IgG1, IgG2, IgG3 or IgG4 (SIGMA®, Saint Louis, MO, USA) diluted to final concentrations of 1.5–0.5 µg/ml in a coating buffer containing 16 mM Na_2_CO_3_ and 35 mM NaHCO_3,_ pH 9.6 and kept at 4°C overnight. The plates were washed three times with lectin buffer containing 10 mM HEPES, pH 7.5, 0.15 M NaCl, 0.01 mM MnCl_2_, 0.1 mM CaCl_2_ and 0.1% v/v Tween 20 and blocked in the same buffer for one hour at room temperature. In the next step purified IgG fraction (dilution 1∶100) was added and the incubation proceeded for another 2 hours at 37°C. After three washes with lectin buffer, 1 µg/ml biotinylated LCA-lectin was added and incubation continued for 1 hour at 37°C. Following three more washes, 0.1 µg/ml peroxidase-labeled streptavidin (Vector Laboratories) was added and the plate was incubated for 1 hour at 37°C. The color reaction was developed with 0.1 M citric acid monohydrate, 0.1 M Na_2_HPO_4_×2H_2_O buffer pH 4.5 containing 0.012% v/v H_2_O_2 _and 1.7 mM 2,2′-azino-bis(3-ethylbenzthiazoline-6-sulphonic acid) (ABTS). The absorbance was read on a model 550 micro plate reader (BIO-RAD, Hercules, CA, USA) at 415 nm. For detection of binding of human IgG subclasses to FcγRs, the plate was coated with soluble FcγRIIa or FcγRIIb or FcγRIIIa at a concentration 5 µg/ml for 20 hours at 4°C. Next day, the plate was blocked with PBS supplemented with 0.05% v/v Tween 20 (PBST) and 2% w/v bovine serum albumin for 2 hours at room temperature. After this step, the purified IgG subclasses, 0.1 µg of each, were added. The plate was washed three times with PBST after the coating step and between each of the following incubation steps. A peroxidase-conjugated protein G (dilution 1∶5000) (BIO-RAD, Hercules, CA, USA) was used for detection. The color reaction was performed as above. All experiments were made in triplicates.

### Radioactive labeling

Proteins were labelled with 0.2 mCi Na ^125^I (PerkinElmer, Upplands-Väsby, Sweden) using the IODE-BEADS Iodination reagent kit (PIERCE, Rockford, IL, USA) according to the manufacturer's instructions. The unbound radioactivity was removed by desalting the proteins on PD-10 Sepharose (Pharmacia, Sweden). The activity of the labeled proteins was estimated to 4 µCi/µg protein.

### Detection of IgG binding to cells

IgG was purified from human plasma treated with EndoS or PBS as described above and thereafter labeled with ^125^iodine (^125^I). For detection of ^125^I-IgG binding to K562 cells, 2×10^6 ^cells were incubated with 0.5×10^6^ cpm of ^125^I-IgG or ^125^I-deglycosylated IgG for 30 minutes at room temperature. After five washes with PBS and centrifugations at 1000×g for three minutes, the radioactivity of the cell pellets was detected using Wallac Wizard™ 1470 Automatic Gamma Counter (PerkinElmer, Waltham, MA, USA). To evaluate the specificity of the binding of radioactive IgG to K562 cells a control experiment was performed. The cells were incubated with 20 µg human IgG in addition to radioactive IgG during the similar incubation conditions as mentioned above. In another experiment, 1×10^6 ^monocytes were incubated with 0.5×10^6^ cpm of ^125^I-IgG or ^125^I-EndoS treated IgG for 30 minutes at room temperature. After five repeated washes of cells with PBS and final pelleting of cells by centrifugation at 1000×g for five minutes, the cells were resuspended in lysis buffer containing 20 mM Tris-HCl pH 7.4, 0.150 M NaCl, 1% v/v Triton-100 and 0.25% v/v NP40 for ten minutes at 4°C. Next, the samples were centrifuged for ten minutes at 14000×g and supernatants applied on a polyacrylamide gel. After separation, the gel was dried and samples analyzed by phosphoimaging in a Fujix BAS 2000 Bioimaging analyzer (Fujifilm Sverige AB, Stockholm, Sweden). In an experiment where the binding of IgG to cells was analyzed by Western blot, 0.5–1×10^6 ^cells were incubated with plasma treated with either EndoS or buffer (as described above), at 37°C for 1 hour. Afterwards, the cells were washed three times with PBS or RPMI medium, resuspended in 100 µL lysis buffer and the bound IgG in cell lysates analyzed by Western blot.

### Incubation of cells with EndoS

K562 cells or monocytes, 2×10^6 ^and 8×10^6 ^respectively, were incubated with two ml of human plasma for 30 minutes at 37°C. The cells were washed five times with PBS and centrifuged at 1000×g for ten minutes after every wash. EndoS, 40 µg in PBS or PBS alone was added to cells and incubation followed for one hour at 37°C. Cells were washed three times with PBS and resuspended in 100 µL lysis buffer. Samples were centrifuged for five minutes at 14000×g, pellets discarded and supernatants analyzed for IgG and glycan contents using SDS-PAGE and Western blot.

### Slot-blotting analysis

IgG1–4, 0.3, 015 and 0.075 µg of each in PBS were applied to PVDF membranes using a slot-blot apparatus from Schleicher and Schuell, Inc., Kene, NH 03431, USA. The membranes were incubated with PBST and 5% skim milk for 1 hour, washed with PBST and incubated with EndoS or EndoS (E235Q), 0.05 mg/ml in PBST and 5% skim milk for 1 hour. After washing, the membranes were incubated with rabbit EndoS-antiserum and subsequently with peroxidase conjugated goat anti-rabbit antibodies. The color development was made using ABTS as peroxidase substrate. All incubation steps were performed at room temperature.

### Surface plasmon resonance interaction analysis

Receptors, IgGs and deglycosylated IgGs were diluted with 10 mM sodium acetate pH 4 and immobilized via amine coupling to different flow cells of CM5 sensorchips (BIAcore, Uppsala, Sweden). Immobilization levels were optimized to around 8000–10000 response units. After determining EndoS(E235Q) as a non-binder to all deglycosylated IgG variants, these flow cells were considered as controls for bulk refraction index changes for EndoS(E235Q) binding to IgG1 throughout IgG4, respectively. In experiments determining IgG1-IgG4 affinity for the receptors FcγRIIa, FcγRIIb and FcγRIIIa, a flow cell subjected to the immobilization protocol but without addition of protein was used as control. For affinity measurements, the binding and dissociation phases were monitored in a BIAcore 2000 instrument. In control experiments for possible mass transfer limitations, the IgGs were injected over the receptors and the EndoS variants over the IgG sub-classes at different flow rates. No differences in initial binding were observed at 5 µl/min or above indicating no limitations to any combinations. Interactants were injected in different concentrations (typically 10–1.25 µg/ml) at 35 µl/min and 25°C over the different coated surfaces (flow cells) (in running buffer: 10 mM HEPES, pH 7.5, 150 mM NaCl, 0.005% surfactant P20, and 3.4 mM EDTA). Between experiments, the surfaces were strictly regenerated with pulses of running buffer containing 2 M NaCl followed by an extensive wash procedure after reaching baseline. For EndoS digestion of IgG bound to pre-immobilized FcγRIIa, an IgG1 concentration (10 µg/ml) was chosen to give a suitable steady-state dissociation phase at a time point were the IgG1 injection was aborted and replaced by running buffer. This experiment was considered as a control and as such compared to an EndoS injection at the same time point after IgG1 binding to FcγRIIa. After X and Y normalization of data, the blank curves from control flow cells of each injected concentration were subtracted. Where applicable, the association (*k_a_*) and dissociation (*k_d_*) rate constants were determined simultaneously using the equation for 1∶1 Langmuir binding in the BIA Evaluation 4.1 software (BIAcore). The binding curves were fitted locally and the equilibrium dissociation constants (*K_D_*) were calculated from mean values of the obtained rate constants.

### Flow cytometry analysis of whole blood

A volume of 15 ml blood was incubated with 0.4 mg EndoS or PBS for 35 minutes at 37°C. An activator of leukocytes, formyl-methionyl-leucyl-phenylalanine (fMLP), (dilution 1∶10000) (SIGMA, Saint Louise, MO, USA) was then added and the incubation continued for 10 minutes at 37°C. Next, blood samples were centrifuged 1000×*g*, five minutes. Plasma and buffy coat were transferred to another tube and centrifuged for five minutes at 1000×*g*. The cells were then washed three times with HBSS containing 30% v/v RPMI and finally resuspended in 100 µl of the same medium. Monoclonal mouse anti-human IgG was prepared by mixing equal amounts of mouse anti-human IgG1 (51 mg/ml), IgG2 (22 mg/ml), IgG3 (16 mg/ml) and IgG4 (24 mg/ml). Five µl of this mixture was added and samples incubated for ten minutes at room temperature. In the next step 5 µl of FITC-conjugated goat anti-mouse IgG (DakoCytomation, Glostrup, Denmark) was added before erythrocytes were lysed using the DakoCytomation Uti-Lyse erythrocyte kit (Carpinteria, CA). Signals were analyzed on a FACSCalibur flow cytometer (Becton Dickinson, Franklin Lakes, NJ, USA). Monocytes were identified by forward scatter and side scatter characteristics (FSC/SSC).

### SDS-PAGE and Western blot analysis

Sodium dodecyl sulfate-polyacrylamide gel electrophoresis (SDS-PAGE) was performed using Mini Protean II cell equipment from BIO-RAD (Hercules, CA, USA) or equipment from LKB (Bromma, Sweden) using the buffer system described by Laemmli [35]. Samples were mixed 1∶1 (v/v) with sample buffer supplemented with 5% mercaptoethanol, and boiled for five minutes before loading onto the 10% polyacrylamide gel. PageRuler™ Protein Ladder Plus (Fermentas, Burlington, Canada) was used as high-molecular-mass standards. The polyacrylamide gels were stained with Coomassie Brilliant Blue R-250 and in some cases dried. For immunoblotting, the gels were transferred to polyvinylidenefluoride (PVDF) membranes (Immobilon P, Millipore, Bedford, MA) as described by Matsudaira (18). After blotting, membranes were blocked in PBS supplemented with 0.05% v/v Tween 20 (PBST) and 5% w/v skim milk (DIFCO, Detroit, MI) for 20 minutes at room temperature. For detection of IgG, the blots were subsequently washed in PBST and then incubated with rabbit anti-human IgG (diluted 1∶3000) (BIO-RAD, Hercules, CA) for one hour at 37°C. After a washing step, membranes were incubated with horseradish peroxidase-conjugated goat anti-rabbit IgG (BIO-RAD) (dilution 1∶1000). For lectin blot analysis, membranes were blocked for 20 minutes in lectin buffer (10 mM HEPES, pH 7.5, 0.15 M NaCl, 0.01 mM MnCl_2_, 0.1 mM CaCl_2_ and 0.1% v/v Tween 20) at room temperature and incubated with biotinylated LCA lectin (diluted 1∶5000). After repeated washes in lectin buffer the membranes were incubated with peroxidase-labeled streptavidin (Vector Laboratories) (diluted 1∶10000). All membranes were developed using SuperSignal West Pico (PIERCE, Rockford, IL) according to the manufacturer's instructions before analyzing by the Chemidoc XRS imaging system and Quantity One image analysis software (BIO-RAD).

## References

[1] Ravetch JV, Bolland S (2001). IgG Fc receptors.. Annu Rev Immunol.

[2] Burton DR, Woof JM (1992). Human antibody effector function.. Adv Immunol.

[3] Arnold JN, Wormald MR, Sim RB, Rudd PM, Dwek RA (2007). The impact of glycosylation on the biological function and structure of human immunoglobulins.. Annu Rev Immunol.

[4] Jefferis R, Lund J, Pound JD (1998). IgG-Fc-mediated effector functions: molecular definition of interaction sites for effector ligands and the role of glycosylation.. Immunol Rev.

[5] Dube R, Rook GA, Steele J, Brealey R, Dwek R (1990). Agalactosyl IgG in inflammatory bowel disease: correlation with C-reactive protein.. Gut.

[6] Parekh RB, Dwek RA, Sutton BJ, Fernandes DL, Leung A (1985). Association of rheumatoid arthritis and primary osteoarthritis with changes in the glycosylation pattern of total serum IgG.. Nature.

[7] Parekh R, Isenberg D, Rook G, Roitt I, Dwek R (1989). A comparative analysis of disease-associated changes in the galactosylation of serum IgG.. J Autoimmun.

[8] van Zeben D, Rook GA, Hazes JM, Zwinderman AH, Zhang Y (1994). Early agalactosylation of IgG is associated with a more progressive disease course in patients with rheumatoid arthritis: results of a follow-up study.. Br J Rheumatol.

[9] Rademacher TW, Williams P, Dwek RA (1994). Agalactosyl glycoforms of IgG autoantibodies are pathogenic.. Proc Natl Acad Sci U S A.

[10] Nimmerjahn F (2006). Activating and inhibitory FcgammaRs in autoimmune disorders.. Springer Semin Immunopathol.

[11] Holland M, Takada K, Okumoto T, Takahashi N, Kato K (2002). Hypogalactosylation of serum IgG in patients with ANCA-associated systemic vasculitis.. Clin Exp Immunol.

[12] Matsumoto A, Shikata K, Takeuchi F, Kojima N, Mizuochi T (2000). Autoantibody activity of IgG rheumatoid factor increases with decreasing levels of galactosylation and sialylation.. J Biochem (Tokyo).

[13] Kaneko Y, Nimmerjahn F, Ravetch JV (2006). Anti-inflammatory activity of immunoglobulin G resulting from Fc sialylation.. Science.

[14] Jefferis R, Lund J (2002). Interaction sites on human IgG-Fc for FcgammaR: current models.. Immunol Lett.

[15] Nimmerjahn F, Ravetch JV (2005). Divergent immunoglobulin g subclass activity through selective Fc receptor binding.. Science.

[16] Woof JM, Burton DR (2004). Human antibody-Fc receptor interactions illuminated by crystal structures.. Nat Rev Immunol.

[17] Nimmerjahn F, Ravetch JV (2006). Fcgamma receptors: old friends and new family members.. Immunity.

[18] Hulett MD, Hogarth PM (1994). Molecular basis of Fc receptor function.. Adv Immunol.

[19] Hulett MD, Witort E, Brinkworth RI, McKenzie IF, Hogarth PM (1994). Identification of the IgG binding site of the human low affinity receptor for IgG Fc gamma RII. Enhancement and ablation of binding by site-directed mutagenesis.. J Biol Chem.

[20] Bisno AL, Stevens DL (1996). Streptococcal infections of skin and soft tissues.. N Engl J Med.

[21] Cunningham MW (2000). Pathogenesis of group A streptococcal infections.. Clin Microbiol Rev.

[22] Fischetti VA (1989). Streptococcal M protein: molecular design and biological behavior.. Clin Microbiol Rev.

[23] Collin M, Olsén A (2001). Effect of SpeB and EndoS from *Streptococcus pyogenes* on human immunoglobulins.. Infect Immun.

[24] Collin M, Olsén A (2001). EndoS, a novel secreted protein from *Streptococcus pyogenes* with endoglycosidase activity on human IgG.. EMBO J.

[25] Collin M, Olsén A (2003). Extracellular enzymes with immuno-modulating activities: variations on a theme in *Streptococcus pyogenes*.. Infect Immun.

[26] Collin M, Svensson MD, Sjöholm AG, Jensenius JC, Sjöbring U (2002). EndoS and SpeB from *Streptococcus pyogenes* inhibit immunoglobulin-mediated opsonophagocytosis.. Infect Immun.

[27] Tarentino AL, Plummer TH (1994). Enzymatic deglycosylation of asparagine-linked glycans: purification, properties, and specificity of oligosaccharide-cleaving enzymes from *Flavobacterium meningosepticum*.. Methods Enzymol.

[28] Collin M, Fischetti VA (2004). A novel secreted endoglycosidase from *Enterococcus faecalis* with activity on human immunoglobulin G and ribonuclease B.. J Biol Chem.

[29] Allhorn M, Olsén A, Collin M (2007). EndoS from *Streptococcus pyogenes* is hydrolyzed by the cysteine proteinase SpeB and requires glutamic acid 235 and tryptophans for IgG glycan-hydrolyzing activity. BMC Microbiol 7: Accepted for publication..

[30] Littaua R, Kurane I, Ennis FA (1990). Human IgG Fc receptor II mediates antibody-dependent enhancement of dengue virus infection.. J Immunol.

[31] Nandakumar KS, Collin M, Olsén A, Nimmerjahn F, Blom AM (2007). Endoglycosidase treatment abrogates IgG arthritogenicity: Importance of IgG glycosylation in arthritis.. Eur J Immunol.

[32] Sjöbring U, Björck L, Kastern W (1991). Streptococcal protein G. Gene structure and protein binding properties.. J Biol Chem.

[33] Forsgren A, Sjöquist J (1966). “Protein A” from *S. aureus*. I. Pseudo-immune reaction with human gamma-globulin.. J Immunol.

[34] Nimmerjahn F, Bruhns P, Horiuchi K, Ravetch JV (2005). FcgammaRIV: a novel FcR with distinct IgG subclass specificity.. Immunity.

[35] Laemmli UK (1970). Cleavage of structural proteins during the assembly of the head of bacteriophage T4.. Nature.

